# Rare case of Pott's puffy tumor due to nasal foreign body

**DOI:** 10.22088/cjim.15.3.553

**Published:** 2024-08-01

**Authors:** Mohsen Mohammadi, Haniyeh Esfandiar, Nazanin Saeidizand, Ali Shaabani, Firooze Esmaeilzadeh

**Affiliations:** 1 Non-Communicable Pediatric Diseases Research Center, Health Research Institute, Babol University of Medical Sciences, Babol, Iran

**Keywords:** Pott’s puffy tumor, Sinusitis, Foreign body, Ear, Nose and Throat

## Abstract

**Background:**

Pott’s Puffy tumor (PPT) is a complicated frontal sinusitis that is also described as frontal bone osteomyelitis and a localized sub periosteal abscess. Early diagnosis and immediate active treatment are necessary to prevent severe neurologic sequelae.

**Case Presentation:**

Here, we report on a case of Pott’s puffy tumor in a previously healthy 13-year-old girl with a worsening headache and swelling of the frontal bone accompanied by vomiting and fever. Magnetic resonance imaging revealed osteomyelitis of the frontal bone. A foreign body was discovered and removed via endoscopy which was then followed by 6 weeks of parenteral and enteral antibiotic therapy, resulting in resolution of all symptoms.

**Conclusion:**

A complete history in terms of trauma and insect bite or foreign body in the nose is paramount for a correct diagnosis, and performing a full examination of ear, nose and throat (ENT) to rule out a foreign body is advised.

Osteomyelitis of the frontal bone is also known as Pott’s puffy tumor. Pott’s puffy tumor manifests as a progressive headache, soft-tissue swelling and fever. Potential complications include meningitis, sagittal vein thrombosis and intracerebral abscesses (1, 2). It usually occurs as a complication of frontal sinusitis and direct trauma to the frontal bone.

The paranasal sinuses are air-filled extensions of the nasal cavity. There are four paired sinuses – named according to the bone in which they are located – maxillary, frontal, sphenoid and ethmoid. Each sinus is lined by a ciliated pseudo stratified epithelium, interspersed with mucus-secreting goblet cells. Frontal sinuses Sensation is supplied by the supraorbital nerve (a branch of the ophthalmic nerve), and arterial supply is via the anterior ethmoidal artery (a branch of the internal carotid). The anatomic location and venous drainage pattern of the frontal sinus can lead to severe and life-threatening extensions of frontal sinus infections. Complications most commonly involve intracranial structures, but can involve the orbit and adjacent bony and soft tissue structures. With the exception of mucoceles, the major complications of frontal sinusitis are infectious.

Adolescence is common age of sinusitis and major risk factors are: Allergic rhinitis or hay fever, Cystic fibrosis, going to daycare, diseases that prevent the cilia from working properly, changes in altitude (flying or scuba diving), large adenoids, smoking (or being second smoker). Weakened immune system from HIV or chemotherapy, abnormal sinus structures. Diagnosis is based on CT scans and MRIs and treatment includes antibiotic therapy and surgical intervention.

We report a case of Pott’s puffy tumor, based on our investigations, the only possible cause for the patient’s condition was the nasal foreign body that is the uniqueness of our report. It confirms the importance of a complete ENT examination.

## Case Presentation

A 13-year-old previously healthy girl was referred to the emergency department with a chief complaint of a gradually worsening frontal headache and acute vomiting, from which she had been suffering for approximately one week. She had been prescribed non-steroid anti-inflammatory medication by her primary care physician. 

**Figure 1:**
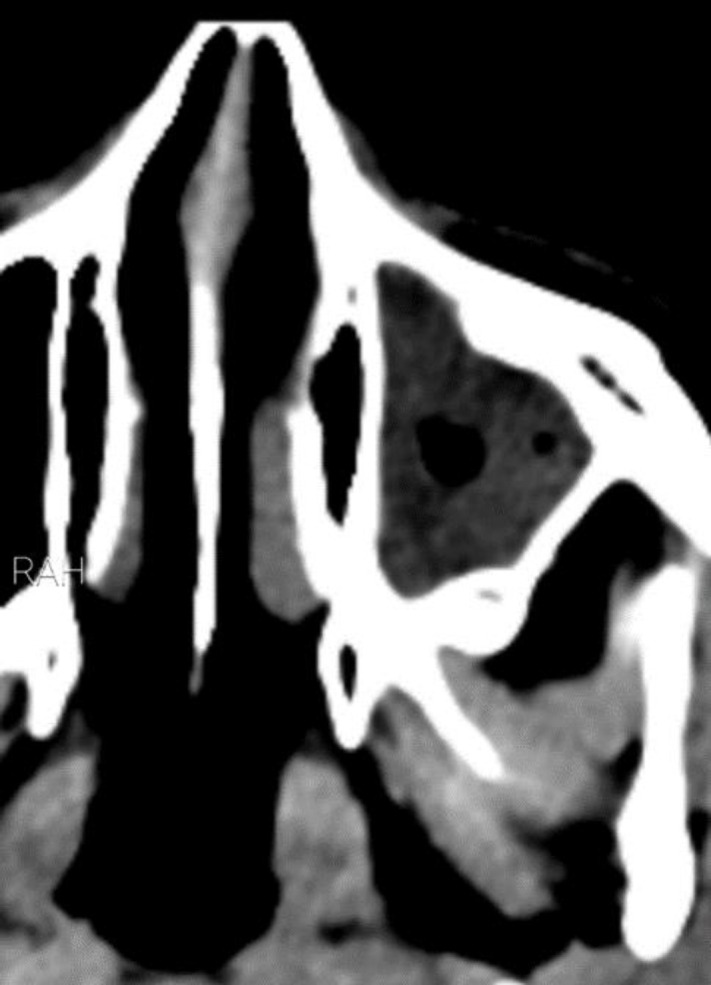
Contrast enhanced computed tomographic scan of head showed right maxillary sinus opacification and right nose fullness

Parents noticed a slowly enlarging soft-tissue swelling over the frontal bone, and she also complained of occasional headaches, which had intensified over the course of the disease. Symptoms accompanied by vomiting and fever, No mood or behavioral changes were noted. Her past medical history was unnotable. She presented with a blood pressure of 110/80 mmHg, a pulse rate of 85 bpm, respiratory rate of 20/min and a temperature of 37.71C. Upon physical examination, she was alert and fully oriented. She was in moderate pain distress. She had a 3x2cm tender, firm, fixed and non-erythematous swelling on the forehead. ENT exam were unremarkable. Neurological examination was normal, with no signs of nuchal rigidity or cervical lymphadenopathy. Other examinations were normal.She had no history of head trauma, recent upper respiratory tract infection or sinopulmonary disease and had had no history of recurrent sinusitis. Our patient and her parents did not mention any history of putting a foreign object in the nose ‘ear or throat. Laboratory blood values showed a 9900 leukocyte count and erythrocyte sedimentation rate and C-reactive protein levels were both elevated, measuring 70mm/h and 50 mg/l respectively. A computed tomographic (CT) scan of the head and orbits and paranasal sinus with intravenous contrast enhancement showed opacification of the right nasal cavity and right ethmoid and maxillary sinuses (figure 1).

**Figure 2:**
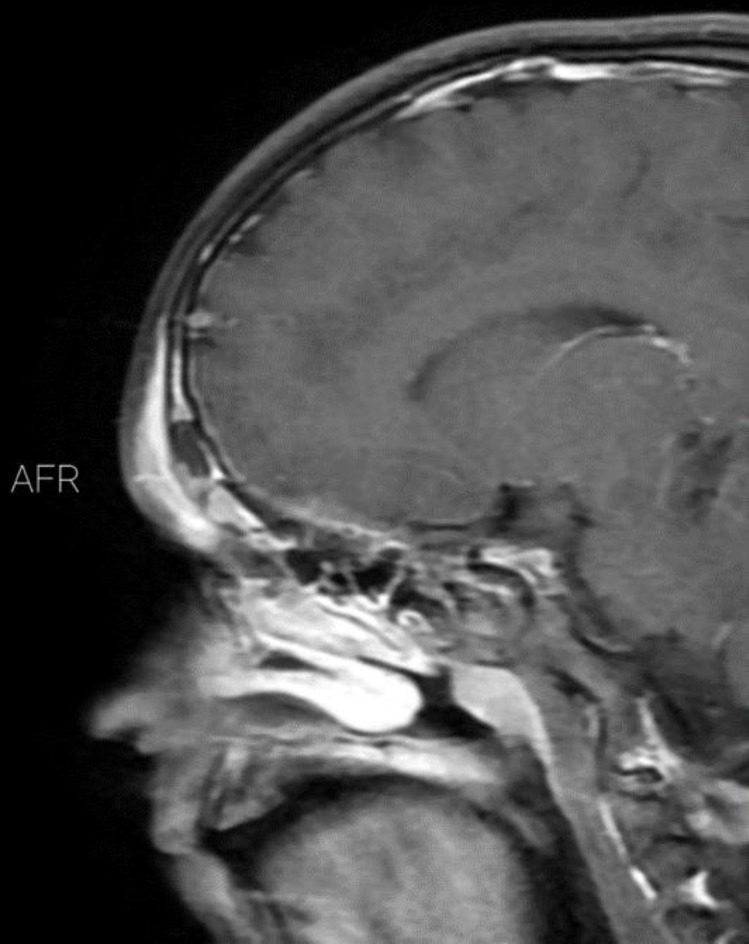
Sagittal T1 magnetic resonance imaging of the head showed mass in frontal sinus and inflammation in soft tissue of forehead

Magnetic resonance imaging of the head showed inflammation of the frontal sinuses (figure 2). The inflammatory changes were seen within the forehead tissue with no intracranial extension. A preoperative diagnosis of Pott’s puffy tumor was confirmed and the patient was administered intravenous ceftriaxone, metronidazole and vancomycin. The patient then underwent a nasal endoscopy and during endoscopic sinus surgery a foreign body that was found to be a date kernel was removed. Unfortunately, the discharged secretions were not studied by smear and culture. During hospitalization patient had frequent vomiting that was controlled with conservative treatment and did not have an organic etiology. The patient was discharged after 10 days of treatment with intravenous metronidazole, vancomycin and ceftriaxone which was then followed by oral cefuroxime and clindamycin.

## Discussion

Pott’s puffy tumor (PPT) is a complicated frontal sinusitis described as frontal bone osteomyelitis and localized subperiosteal abscess. This condition can be diagnosed with the classical presentation of headache, fever and swelling over the forehead and upper eyelids (3). Patients are expected to have some degree of immune deficiency or a history of recurrent sinusitis. It usually occurs as a complication of frontal sinusitis and/or direct trauma to the frontal bone (4); PPT developing following insect bite has also been described (5). 

This patient, had no history of head trauma, recent upper respiratory tract infection or sinopulmonary disease and had no history of recurrent sinusitis and based on our investigations, the only possible cause for the patient’s condition was the nasal foreign body that is the uniqueness of our report.

Clinical features of Pott’s puffy tumor include a soft-tissue swelling of the forehead, fever,headache, vomiting and nasal symptoms (6). An acute or progressive headache may be an expression of an intracranial Invasion (7). Overuse of antibiotics hides classic clinical features of Pott's puffy tumor. The presentation may be subtle as in the case with our patient, in which there was no recent upper respiratory tract infection or sinopulmonary disease and no history of recurrent sinusitis. Although, our patient reported slight and soft swelling of the forehead with imperceptible forehead protrusion and recurrent vomiting which persisted into the early days of treatment. She also had a high fever which was easily controlled with medication. Cased Pellejero et al. reported an exceptionally unusual case of a PPT of the frontal bone after blunt trauma (closed head injury), with an intracranial infected lesion whereby Actinomyces was isolated after surgery, along with Fusobacterium and Propionibacterium (4).

V Raja et al. reported a healthy 49-year-old Caucasian male presenting with swelling of the right upper eyelid of one-week duration following an insect bite. There was no previous history of ocular infection or any other medical problems. The case demonstrates the aggressive nature of this condition and its resistance to intensive systemic antibiotics. The patient's condition improved only after thorough surgical drainage (5).

Diagnostic measures after history taking and physical examination includes a CT-Scan of the brain and PNS, which is a choice study for Pott’s puffy tumor and MRI with contrast Venus media that can show invasion of disease to brain parenchyma; also, bone nuclear scan allows early detection of osteomyelitis (2). In this case, after a CT scan that showed inflammatory involvement of the right ethmoid and maxillary sinuses, Pott’s puffy tumor was suspected and we decided to do an MRI contrast study that showed frontal sinusitis, frontal bone osteomyelitis and inflammation of soft tissue of the forehead.

In spite of the results of the ENT examination and a negative history for possible presence of a foreign body, and based on clinical suspicion, we performed an intranasal endoscopic imaging.

The definitive management of Pott’s puffy tumor includes a combination of antibiotics and surgical therapy to prevent further penetration of introduction of the infection into the cranium (7, 8). Percutaneous frontal sinus trephination combined with endoscopic sinus surgery has been described in the literature as a successful procedure when treating Pott’s puffy tumor secondary to frontal sinus infection. In addition to surgical drainage of the abscess and debridement of osteomyelitis an empiric antibiotic regimen should provide coverage against Streptococci, Staphylococcus aurous and anaerobes, it can also adequately penetrate the blood-brain barrier (9). As with our patient, who had had no history of recent sinusitis, with the endoscopic sinus surgery that was initially aimed towards debridement and drainage, a foreign body—date kernel—was discovered and extracted (7). Patient fully recovered with no need for further surgical treatment, and with a 6-week antibiotic regimen that includes 10 days of intravenous metronidazole, vancomycin and ceftriaxone followed by oral cefuroxime and clindamycin. The patient gets better and headache and frontal swelling are reduced.

A 13-year-old girl, presenting with worsening headache, vomiting, fever and chills who had no recent upper respiratory tract infection or a history of recurrent sinusitis and sinopulmonary disease was diagnosed with Pott’s puffy tumor with computerized tomography and magnetic resonance imaging. 

Despite the normal ENT examination and lack of any reports of foreign body introduction into the upper respiratory tract, during the surgical debridement of the infected areas of the sinuses, a foreign body was discovered. After antibiotic therapy, the patient achieved compete recovery.

As a result, although Pott’s puffy tumor is a rare creature, it is a serious consequence of frontal sinusitis or direct trauma. It may have no symptoms, which would leave it untreated, increasing the chance of life-threatening complications of the nervous system. Get a complete history in terms of trauma and foreign body in the nose or insect bites, performing a full examination of the ear, nose and throat for the foreign body is recommended.
